# Knowledge, Concerns, and Behaviors of Individuals During the First Week of the Coronavirus Disease 2019 Pandemic in Italy

**DOI:** 10.1001/jamanetworkopen.2020.15821

**Published:** 2020-07-24

**Authors:** Francesco Pagnini, Andrea Bonanomi, Semira Tagliabue, Michela Balconi, Mauro Bertolotti, Emanuela Confalonieri, Cinzia Di Dio, Gabriella Gilli, Guendalina Graffigna, Camillo Regalia, Emanuela Saita, Daniela Villani

**Affiliations:** 1 Department of Psychology, Università Cattolica del Sacro Cuore, Milan, Italy; 2 Department of Psychology, Harvard University, Cambridge, Massachusetts; 3 Department of Statistical Sciences, Università Cattolica del Sacro Cuore, Milan, Italy"; 4 EngageMinds HUB, Consumer, Food & Health Engagement Research Center, Università Cattolica del Sacro Cuore, Milan, Italy

## Abstract

**Importance:**

At the beginning of a public health crisis, such as the coronavirus disease 2019 (COVID-19) pandemic, it is important to collect information about people’s knowledge, worries, and behaviors to examine their influence on quality of life and to understand individual characteristics associated with these reactions. Such information could help to guide health authorities in providing informed interventions and clear communications.

**Objectives:**

To document the initial knowledge about COVID-19 and recommended health behaviors; to assess worries (ie, one’s perception of the influence of the worries of others on oneself), social appraisal, and preventive behaviors, comparing respondents from areas under different movement restrictions during the first week after the outbreak; and to understand how worries, perceived risk, and preventive behaviors were associated with quality of life and individual characteristics among Italian adults.

**Design, Setting, and Participants:**

This convenience sample, nonprobablistic survey study recruited adult participants with a snowballing sampling method in any Italian region during the first week of the COVID-19 outbreak in Italy from February 26, 2020, to March 4, 2020. Data were analyzed from March 5 to 12, 2020.

**Exposures:**

Information was collected from citizens living in the quarantine zone (ie, red zone), area with restricted movements (ie, yellow zone), and COVID-19–free regions (ie, green zone).

**Main Outcomes and Measures:**

Levels of knowledge on the virus, contagion-related worries, social appraisal, and preventive behaviors were assessed with ratings of quality of life (measured using the Short Form Health Survey). Additionally, some individual characteristics that may be associated with worries and behaviors were assessed, including demographic characteristics, personality traits (measured using Big Five Inventory-10), perceived health control (measured using the internal control measure in the Health Locus of Control scale), optimism (measured using the Revised Life Orientation Test), and the need for cognitive closure (measured using the Need for Closure Scale).

**Results:**

A total of 3109 individuals accessed the online questionnaire, and 2886 individuals responded to the questionnaire at least partially (mean [SD] age, 30.7 [13.2] years; 2203 [76.3%] women). Most participants were well informed about the virus characteristics and suggested behaviors, with a mean (SD) score of 77.4% (17.3%) correct answers. Quality of life was similar across the 3 zones (effect size = 0.02), but mental health was negatively associated with contagion-related worries (β = –0.066), social appraisal (β = –0.221), and preventive behaviors (β = –0.066) in the yellow zone (*R*^2^ = 0.108). Social appraisal was also associated with reduced psychological well-being in the green zone (β = –0.205; *R*^2^ = 0.121). In the yellow zone, higher worries were negatively correlated with emotional stability (β = –0.165; *R*^2^ = 0.047). Emotional stability was also negatively associated with perceived susceptibility in the yellow (β = –0.108; *R*^2^ = 0.040) and green (β = –0.170; *R*^2^ = 0.087) zones. Preventative behaviors and social appraisal were also associated with the need for cognitive closure in both yellow (preventive behavior: β = 0.110; *R*^2^ = 0.023; social appraisal β = 0.115; *R*^2^ = 0.104) and green (preventive behavior: β = 0.174; *R*^2^ = 0.022; social appraisal: 0.261; *R*^2^ = 0.137) zones.

**Conclusions and Relevance:**

These findings suggest that during the first week of the COVID-19 outbreak in Italy, people were well informed and had a relatively stable level of worries. Quality of life did not vary across the areas, although mental well-being was challenged by the social appraisal and worries related to the contagion. Increased scores for worries and concerns were associated with more cognitive rigidity and emotional instability.

## Key Points

**Question:**

What were the worries and perceptions experienced by residents of different exposure areas during the first week of outbreak of the coronavirus disease 2019 (COVID-19) in Italy?

**Findings:**

This survey study including 2886 participants found that people were well informed about COVID-19 and its implications. Higher scores for cognitive rigidity and emotional instability were associated with more worries and concerns regarding the COVID-19 outbreak regardless of exposure region.

**Meaning:**

These findings suggest that at the beginning of the COVID-19 outbreak, people who were cognitively flexible and emotionally stable were more likely to be more resilient to worries and concerns relating to COVID-19.

## Teaser

This survey study examined the knowledge, concerns, and behaviors of individuals living in different coronavirus disease 2019 (COVID-19) exposure zones during the first week of the pandemic in Italy.

## Introduction

The coronavirus disease 2019 (COVID-19) pandemic, which began in China in December 2019, has spread to many other countries in the world, and it is considered a public health emergency of international concern.^1^ Fever and cough are the most common symptoms, and severe respiratory problems occur in approximately 15% of cases.^2^ As of March 20, 2020, approximately 110 000 people were infected with severe acute respiratory syndrome coronavirus 2 (SARS-CoV-2), the virus responsible for COVID-19, in 105 countries around the world.^3^ Together with the medical and epidemiological issues, this emergency situation has several psychological and social effects.^4^ These effects on psychological well-being tend to get worse with time, especially for individuals whose freedom has been significantly restricted.^5^

Risk perception and worries about the contagion are psychological aspects that are particularly relevant in epidemic or pandemic scenarios, as they shape social reactions and behavior changes.^6^ These psychological processes are activated by the fear of uncertainty and uncontrollability, and they tend to be associated with an exaggerated estimation of the real risk of infection.^7^ These reactions are fairly heterogeneous among people, but they are associated with certain demographic variables and individual characteristics.^8^ For example, men tend to perceive lower levels of risk compared with women.^9^

As for the individual characteristics, one domain that is beginning to receive considerable attention is the intersection between disease avoidance processes and personality traits.^10,11^ Personality traits are relatively stable characteristics that organize and guide social beliefs and behavior. One well-known model describing personality structure is the 5-factor model,^12^ encompassing the traits of extraversion, agreeableness, conscientiousness, neuroticism, and openness to experience. Specifically, individuals high in neuroticism are typically triggered by stressors, such as threat and uncertainty,^13^ while those high in openness take advantage of their flexibility and creativity to cope better with stress,^14^ and those high in consciousness are more prone to engage in healthy behaviors.^15^

Furthermore, the literature suggests that individual characteristics associated with coping with uncertainty, such as dispositional intolerance for uncertainty, scarce optimism, and a low level of perceived control, can negatively influence the psychological adaptation process in emergency situations.^16,17,18^ The geographic proximity to a threat is another factor that intuitively enhances concerns and perceived risks, increasing the perception of vulnerability.^19^

The Italian COVID-19 outbreak is a peculiar scenario, as Italy rapidly became the country with the second most SARS-CoV-2 infections by number, which was more than 15 000 infections as of March 20, 2020, In an effort to contain the virus before the entire country was put on lockdown, Italian health authorities established a quarantine zone around 10 municipalities where the outbreak had begun (known as the *red zone*) from February 22 to March 3, 2020. During this period, schools were shut down and traveling and commuting were strongly discouraged in the surrounding areas (known as the *yellow zone*), resulting in important lifestyle changes in several Italian regions, especially Lombardy and Veneto.^20^ Meanwhile, no restrictions were imposed on the other regions (known as the *green zone*). These new social rules and norms (eg, to not stand closer than within 1 m of another person), together with the uncertainty and uncontrollability of the situation, may confuse or scare people who may already be afraid of the perceived risk of being exposed to SARS-CoV-2. Furthermore, fear and anxiety can easily be spread through a social appraisal, which is the main mechanism for the contagion of emotions.^21^

While Italy faced this situation, other countries in Europe and in the rest of the world have also faced or may yet face similar challenges in the near future, as the spread of the virus continues. For this reason, we considered it important to monitor and assess the levels of information and awareness, together with the worries for the spread of the virus, those related to the social appraisal of the pandemic, and the contagion-related behaviors experienced by people in Italy. Together with the assessment of these beliefs, we were interested in documenting the levels of quality of life during this early stage of the pandemic and investigating whether some individual characteristics were associated with concerns and behaviors.

Specifically, this study had 3 aims. The first aim was to document individuals’ knowledge about COVID-19 and the recommended health behaviors and to assess worries, social appraisal, and preventive behaviors, comparing respondents from the quarantine area (red zone), the rest of the regions undergoing freedom-restrictive rules (yellow zone), and the COVID-19–free regions (green zone). Given the rapid diffusion of COVID-19 in a specific area of the north of Italy (ie, the quarantine area), we anticipated that residents of this area would experience higher levels of worries and risk perception than individuals in other parts of Italy. The second aim was to assess the associations of the pandemic and contagion-related worries with quality of life during the first week of the COVID-19 outbreak in the populations from the 3 geographical areas. The third aim was to investigate the role of a set of individual characteristics as possible risk factors associated with worries, perceived risk, and preventive behaviors.

## Methods

This survey study was approved by the ethics commission of the Department of Psychology at Università Cattolica del Sacro Cuore. All participants provided written informed consent. The sample for this survey study was self-selected and therefore nonprobabilistic, preventing use of protocols to quantify invitations and response rates, according to the American Association for Public Opinion Research (AAPOR) reporting guideline.

This survey study was conducted online. The sample was obtained with a snowballing sampling method and by posting the invitation to join the study on Facebook (and to a lesser extent, on Instagram) (Facebook) with particular efforts to reach participants from the red zone (eg, posting on Facebook local groups). The inclusion criteria were being aged 18 years or older and being a resident of any Italian region. The survey was delivered through the Qualtrics online survey suite (Qualtrics), and remained available for 1 week, from February 26 to March 4, 2020. All questions and questionnaires were given in Italian.

### Epidemic Knowledge, Worries, and Intended Behaviors

Together with demographic characteristic data, we collected information about the participants’ knowledge on COVID-19 based on the recommendations provided by the Italian Ministry of Health at the time when data were collected. Participants read 10 sentences, such as “Coronavirus can be transmitted from person to person” or “A surgical mask prevents healthy people from contracting coronavirus,” and were asked to indicate whether the statement was true, false, or whether the respondent did not know. Furthermore, we assessed pandemic-related worries (ie, how worried this person is about the COVID-19–related situation), perceived susceptibility (ie, what are the odds for this person to contract COVID-19), social appraisal (eg, how much did this person feel they were being affected by the worries of other people), and preventive behaviors (eg, was this person limiting contact with others?) with 8 Likert-scale items. These items have been grouped into 4 different measures: pandemic-related worries (item 1), perceived susceptibility (item 2), preventive behaviors (items 3 and 4; mean score of 2 items, *r* = 0.544), and social appraisal (items 5, 6, 7, and 8; mean score of 4 items, α = .857). The full list of worries and their frequencies is reported in eTable 1 in the Supplement.

### Quality of Life

Quality of life was assessed with the Short Form Health Survey,^22^ which is a widely used and psychometrically reliable instrument. The Short Form Health Survey comprised 12 items that provide information about 2 health-related quality of life domains: physical health (measured using the Physical Composite Scale) and mental health (measured using the Mental Composite Scale). Scores range from 0 to 100, with higher scores indicating better health.

### Individual Characteristics

We assessed personality traits with the short version of the Big Five Inventory-10,^23^ a reliable instrument that provides the scores for extraversion (eg, being extraverted and enthusiastic), agreeableness (eg, being sympathetic and warm), conscientiousness (eg, being organized and self-disciplined), emotional stability (eg, being calm, stable, and balanced), and openness (eg, being creative and open to new experiences). The need for cognitive closure (ie, the need for definite answers, order, and closure and an aversion for ambiguity) was measured using a 6-item scale^24^ adapted from the Need for Closure scale.^25^ Optimism was measured with the Revised Life Orientation Test,^26^ a 10-item questionnaire that assesses individual differences in generalized optimism vs pessimism. The factor for internal control from the Health Locus of Control scale,^27^ comprising 6 items, was used to measure health-related perceived control, the belief that one has control over one’s own health.

### Statistical Analysis

Descriptive statistics (ie, frequency, mean, and SD) were performed to present findings for each variable measured. Analyses of variance were performed to verify the presence of sex, age, education, and zone differences on all the variables.

To test whether pandemic-related worries and behaviors were associated with physical and mental health (the domains of the Short Form Health Survey), 2 separate hierarchical multiple regression analyses were conducted for each zone for a total of 6 regression models. In each regression, sex and age were included in the first step, and a stepwise regression method was used to determine how worries and behaviors were associated with health in the second step.

Finally, to analyze which psychological variables could be factors associated with worries and behaviors, for each of the zones, 4 separate linear hierarchical multiple regressions were performed for a total of 12 multiple regression models. For each regression, sex and age were included in the first step, while in the second step, a stepwise regression method was used to determine which of the outcomes included (Big Five Inventory-10 traits, need for cognitive closure, Revised Life Orientation Test, and Health Locus of Control subscales) was significantly associated with worries and behaviors. To adjust for the 2-sided significance values, we followed the procedure suggested by Moiseev (ie, formula 6).^28^ In particular, to obtain an adjusted *P* = .05, we used *P* = .0085 for regressions estimating health by worries and behaviors and *P* = .0051 for regressions estimating worries and behaviors by individual characteristics. As not all the participants completed all the items of the survey, there were some missing data. These missing data ranged from 2% (for the pandemic-related worries) to 16% (for the Health Locus of Control scale items). Sociodemographic and psychological variables were checked for random missing data distribution with Little Test for missing completely at random (χ^2^_35_ = 35.7; *P* = .44). Data were analyzed using SPSS statistical software version 25.0 (IBM) from March 5 to 20, 2020.

## Results

### Participants

A total of 3109 individuals accessed the online questionnaire, and 2886 individuals responded to the questionnaire at least partially. Mean (SD) age was 30.7 (13.2) years, and 2203 (76.3%) were women. Regarding education, 3 participants (0.1%) had only completed primary school, 89 participants (3.1%) had completed only middle school, 1327 participants (46.1%) had a high school diploma or equivalent, 656 participants (22.8%) had a bachelor’s degree, 564 participants (19.6%) had a master’s degree, and 241 participants (8.4%) had postgraduate education. Most participants lived in areas with at least some restrictions, including 108 participants (3.7%) in the red zone and 2382 participants (82.5%) in the yellow zone. Descriptive statistics stratified by area are reported in Table 1. The eFigure in the Supplement illustrates the sample distribution on a contagion map.

**Table 1.** Descriptive Statistics and ANOVAs on Each Variable by Area

Variables	Total respondents, No.	Residence Zone, mean (SD)			*P* value	Effect size
		Red	Yellow	Green		
Women, No. (%)	2880	84 (77.8)	1847 (77.5)	268 (67.7)	<.001	0.078
Age, y	2882	34.37 (11.21)	29.79 (12.68)	34.88 (15.42)	<.001	0.144
Education, No. (%)^a^						
Low	93	7 (6.5)	66 (2.7)	20 (5.1)	.01	.049
Medium	1327	48 (44.4)	1119 (47.1)	160 (40.4)		
High	1461	53 (49.1)	1192 (50.2)	216 (54.5)		
Assessment score, mean (SD)						
Physical health	2619	54.45 (5.07)	54.25 (5.69)	53.88 (6.20)	.49	0.023
Mental health	2619	47.18 (10.65)	47.94 (10.39)	47.44 (10.82)	.57	0.020
Optimism	2447	2.73 (0.71)	2.72 (0.81)	2.79 (0.79)	.31	0.031
Need for cognitive closure	2437	3.80 (0.79)	3.81 (0.82)	3.93 (0.83)	.05	0.049
Internal locus of control	2411	3.60 (0.86)	3.72 (0.88)	3.87 (0.89)	.006	0.065
Agreeableness	2428	3.18 (0.85)	3.18 (0.84)	3.24 (0.83)	.40	0.027
Conscientiousness	2428	3.68 (0.72)	3.69 (0.80)	3.72 (0.83)	.86	0.011
Emotional stability	2428	3.07 (1.05)	2.97 (1.01)	2.92 (1.05)	.45	0.025
Extroversion	2428	3.24 (0.86)	3.23 (0.92)	3.19 (0.94)	.76	0.015
Openness	2428	3.39 (1.04)	3.67 (0.92)	3.61 (0.99)	.02	0.056
Epidemic-related worries	2830	3.00 (1.48)	2.99 (1.47)	2.79 (1.56)	.05	0.046
Perceived susceptibility	2829	4.20 (1.43)	3.88 (1.36)	3.57 (1.35)	<.001	0.092
Preventive behaviors	2829	4.25 (1.55)	3.13 (1.34)	2.88 (1.38)	<.001	0.170
Social appraisal	2828	3.44 (1.61)	3.41 (1.52)	3.24 (1.49)	.09	0.041

^a^ Education level was classified as low, primary to middle school; medium, high school diploma; and high, bachelor’s or masters degree or postgraduate degree.

### Knowledge Regarding COVID-19

Participants expressed a good knowledge regarding COVID-19 and recommended behaviors, with a mean (SD) of 77.4% (17.3%) correct answers (Table 2). The statements with the lowest percentage of correct answers were “Antibiotics can help preventing new Coronavirus infections” with 71.9% of participants responding correctly and 11.7% reporting not knowing the answer, and especially, “Coronavirus is spread by contact with bus or subway handles,” with 29.5% of respondents answering correctly and 17.4% reporting not knowing the answer. Regarding spread of COVID-19 via bus or subway handles, the correct answer at the time was false, according to the Italian health authorities, although updated studies^29^ now disprove this information. Owing to the high percentage of participants who correctly answered these items, they were not used in the rest of our analyses.

**Table 2.** Knowledge Regarding Coronavirus Disease 2019

Statement	Answer, No. (%) (n = 2813)		
	True	False	I do not know
Coronavirus can be transmitted from person to person^a^	2338 (83.1)^b^	48 (1.7)	428 (15.2)
Coronavirus can be transmitted through saliva	2607 (92.7)^b^	77 (2.7)	129 (4.6)
Coronavirus is spread by contact with the bus or the subway handles	1493 (53.1)	830 (29.5)^b^	490 (17.4)
Hand washing and disinfection are a key element in preventing infection	2450 (87.1)^b^	207 (7.4)	156 (5.6)
Parcels from China or other countries with a high infection rate can transmit coronavirus	513 (18.2)	2090 (74.3)^b^	210 (7.5)
People can contract coronavirus from their dogs or cats	43 (1.5)	2610 (92.8)^b^	160 (5.7)
A surgical mask prevents healthy people from contracting coronavirus	203 (7.2)	2380 (84.6)^b^	230 (8.2)
A person suspected of having contracted coronavirus, or with symptoms such as coughing or sneezing, should wear the mask in the presence of other people in these days	2257 (80.2)^b^	448 (15.9)	108 (3.8)
Antibiotics can help preventing new coronavirus infections	461 (16.4)	2022 (71.9)^b^	330 (11.7)
In case of symptoms such as cough or fever, people should go to the emergency department	403 (14.3)	2208 (78.5)^b^	202 (7.2)

^a^ Includes 2814 respondents.

^b^ Indicates the correct answer per the Italian health authorities at the time of the survey.

### Worries and Psychological Variables

In all zones, scores for worries and intended behaviors were higher among women than men. Moreover, older participants were less worried about their risk of getting sick, and youngest people reported fewer intentions to enact protective behaviors (eg, maintaining distance from other people or avoiding meeting them), but they seemed more worried to see others around them worried (eTable 2 in the Supplement).

People living in the red zone had higher mean (SD) scores for preventive behaviors than people living in the other 2 zones (red: 4.25 [1.55]; yellow: 3.13 [1.34]; green: 2.88 [1.38]; *P* < .001), whereas people living in the green zone had lower mean (SD) scores for worries about getting sick (red: 4.20 [1.43]; yellow: 3.88 [1.36]; green: 3.57 [1.35]; *P* < .001) (Table 1). There were no appreciable differences for the psychological outcomes among the 3 zones.

### Physical and Mental Health

Quality of life was similar across the 3 areas (effect size = 0.02), but mental health was negatively associated with the contagion-related worries (β = –0.066), social appraisal (β = –0.221), and preventive behaviors (β = –0.066) in the yellow zone (*R*^2^ = 0.108). Mental health scores were associated with most of the worries across the areas (Table 3). Regression models indicated that social appraisal was the strongest risk factor associated with mental health in yellow (β = –0.221; *R*^2^ = 0.108) and green (β = –0.205; *R*^2^ = 0.121) zones (Table 4). In the yellow zone, higher scores for pandemic-related worries (β = –0.064) and preventive behaviors (β = –0.066) were associated with worse mental health (*R*^2^ = 0.108). Physical health was not associated by scores for worries, with the only exception of participants living in the yellow zone, among whom better physical health scores were associated with higher social appraisal scores (β = 0.094) and lower levels of protective behavior (β = −0.079) (Table 4).

**Table 3.** Correlation Matrix Between Physical and Mental Health and Worries and Behaviors for Area

Trait	*R*^2^											
	Epidemic-related worries			Perceived susceptibility			Preventive behaviors			Social appraisal		
	Red^a^	Yellow^b^	Green^c^	Red^a^	Yellow^b^	Green^c^	Red^a^	Yellow^b^	Green^c^	Red^a^	Yellow^b^	Green^c^
Physical health	0.017	0.004	–0.081	0.029	<0.001	–0.018	–0.095	–0.051	–0.081	0.017	0.080	0.030
*P* value	.87	.85	.13	.78	>.99	.73	.35	.02	.13	.87	<.001	.58
Mental health	–0.171	–0.193	–0.133	–0.221	–0.177	–0.220	–0.082	–0.175	–0.117	–0.292	–0.300	–0.262
*P* value	.09	<.001	.01	.03	<.001	<.001	.42	<.001	.03	.003	<.001	<.001
Optimism	0.168	0.073	0.004	0.206	0.118	0.089	0.068	0.066	0.044	0.118	0.141	0.115
*P* value	.12	.001	.94	.06	<.001	.11	.53	.003	.43	.28	<.001	.040
Need for cognitive closure	0.147	0.124	0.146	0.205	0.102	0.154	0.197	0.113	0.173	0.259	0.207	0.286
*P* value	.18	<.001	.009	.06	<.001	.006	.07	<.001	.002	.02	<.001	<.001
Internal locus of control	–0.098	–0.058	0.012	–0.042	–0.013	0.073	–0.027	–0.025	–0.016	0.131	0.004	0.011
*P* value	.38	.01	.83	.71	.56	.20	.81	.27	.77	.24	.87	.85
Agreeableness	–0.085	–0.041	–0.111	0.027	–0.043	–0.086	0.058	–0.048	–0.064	0.061	–0.052	–0.117
*P* value	.44	.06	.049	.81	.065	.13	.60	.03	.26	.58	.02	.04
Conscientiousness	–0.117	–0.008	0.089	–0.196	–0.056	–0.031	–0.040	–0.031	–0.013	–0.023	–0.071	–0.020
*P* value	.29	.71	.11	.07	.01	.58	.72	.17	.82	.83	.001	.72
Emotional stability	–0.293^a^	–0.177	–0.165	–0.066	–0.144	–0.214	0.022	–0.096	–0.118	–0.243	–0.259	–0.228
*P* value	.007	<.001	.003	.55	<.001	<.001	.84	<.001	.04	.03	<.001	<.001
Extroversion	–0.066	–0.056	0.032	0.088	–0.035	0.034	–0.114	–0.079	–0.061	–0.020	–0.038	–0.009
*P* value	.55	.01	.57	.43	.12	.55	.30	<.001	.28	.86	.09	.88
Openness	–0.072	–0.004	–0.043	–0.242	–0.005	0.029	–0.171	–0.010	–0.041	–0.077	–0.039	–0.057
*P* value	.51	.87	.44	.03	.81	.60	.12	.66	.47	.49	.08	.31

^a^ Includes data for 98 respondents.

^b^ Includes data for 2169 respondents.

^c^ Includes data for 351 respondents.

**Table 4.** Regression Model Estimating Mental and Physical Health by Worries and Behaviors^a^

Trait	β	t	*P* value	*R*^2^
**Mental Health**				
Red zone				
Intercept	NA	10.583	<.001	0.027
Sex	–0.071	–0.710	.48	
Age	0.210	2.090	.04	
Yellow zone				
Intercept	NA	58.908	<.001	0.108
Sex	–0.012	–0.563	.57	
Age	0.122	5.836	<.001	
Social appraisal	–0.221	–8.814	<.001	
Preventive behaviors	–0.066	–2.868	.004	
Epidemic-related worries	–0.064	–2.724	.006	
Green zone				
Intercept)	NA	21.316	<.001	0.121
Sex	–0.007	–0.139	.89	
Age	0.251	4.849	<.001	
Social appraisal	–0.205	–3.921	<.001	
**Physical health**				
Red zone				
Intercept	NA	33.312	<.001	0.094
Sex	–0.102	–1.053	.29	
Age	–0.313	–3.228	.002	
Yellow zone				
Intercept	NA	111.958	<.001	0.041
Sex	–0.053	–2.504	.01	
Age	–0.166	–7.680	<.001	
Social appraisal	0.094	3.936	<.001	
Preventive behaviors	–0.079	–3.382	.001	
Green zone				
Intercept	NA	57.530	<.001	0.054
Sex	–0.075	–1.429	.15	
Age	–0.243	–4.607	<.001	

Abbreviation: NA, not applicable.

^a^ Data are adjusted for sex and age.

### Predicting Worries and Protective Behaviors

The role of individual characteristics in estimating worries and behaviors is reported in Table 5. Pandemic-related worries were particularly associated with emotional stability in the yellow zone (β = –0.165; *R*^2^ = 0.047), that is, people with lower emotional stability scores were more likely to expressed more worries. In particular, perceived susceptibility was negatively associated with emotional stability in the yellow (β = –0.108; *R*^2^ = 0.040) and green (β = –0.170; *R*^2^ = 0.087) zones. In the red zone the only significant factor associated with perceived susceptibility was openness (β = –0.307; *R*^2^ = 0.116), that is, participants with the most worries about getting sick had lower levels of openness than less worried participants. Preventive behaviors and social appraisal were not significantly associated with psychological variables in the red zone. Adopting preventive behaviors was associated with higher ratings for need for cognitive closure in the yellow (β = 0.110; *R*^2^ = 0.023) and green (β = 0.174; *R*^2^ = 0.023) zones, and in the yellow zone, people with lower extraversion were also more likely to adopt preventive measures (β = –0.072). Social appraisal ratings were positively associated with the need for cognitive closure in the yellow (β = 0.115; *R*^2^ = 0.104) and green (β = 0.261; *R*^2^ = 0.137) zones and negatively associated with emotional stability scores in the yellow zone (β = –0.182). More specifically, in the green zone, people who reported feeling more influenced by the worries of others had higher scores for need for cognitive closure (β = 0.261).

**Table 5.** Regression Model Estimating Worries and Behaviors by Individual Characteristics^a^

Trait	β	t	*P* value	R2
**Epidemic-related worries**				
Red zone				
Intercept	NA	7.003	<.001	0.056
Sex	0.233	2.126	.03	
Age	–0.087	–0.793	.43	
Yellow zone				
Intercept	NA	27.406	<.001	0.047
Sex	0.123	5.557	<.001	
Age	0.036	1.599	.11	
Emotional stability	–0.165	–7.332	<.001	
Green zone				
Intercept	NA	12.208	<.001	0.042
Sex	0.185	3.305	.001	
Age	–0.064	–1.141	.25	
**Perceived susceptibility**				
Red zone				
Intercept	NA	9.111	<.001	0.116
Sex	–0.015	–0.137	.89	
Age	–0.291	–2.743	.008	
Openness	–0.307	–2.865	.005	
Yellow zone				
Intercept	NA	35.623	<.001	0.040
Sex	0.057	2.560	.01	
Age	–0.134	–5.994	<.001	
Emotional stability	–0.108	–4.815	<.001	
Green zone				
Intercept	NA	16.139	<.001	0.087
Sex	0.082	1.504	.13	
Age	–0.199	–3.612	<.001	
Emotional stability	–0.170	–3.102	.002	
**Preventive behaviors**				
Red zone				
Intercept	NA	5.454	<.001	0.006
Sex	0.093	0.839	.40	
Age	0.134	1.200	.23	
Yellow zone				
Intercept	NA	10.969	<.001	0.023
Sex	0.060	2.675	.008	
Age	0.062	2.766	.006	
Need for cognitive closure	0.110	4.919	<.001	
Extroversion	–0.072	–3.240	.001	
Green zone				
Intercept	NA	3.118	.002	0.022
Sex	0.033	0.589	.56	
Age	0.026	0.456	.65	
Need for cognitive closure	0.174	3.102	.002	
**Social appraisal**				
Red zone				
Intercept	NA	6.983	<.001	0.051
Sex	0.138	1.267	.21	
Age	–0.257	–2.361	.02	
Yellow zone				
Intercept	NA	14.324	<.001	0.104
Sex	0.104	4.818	<.001	
Age	–0.115	–5.340	<.001	
Need for cognitive closure	0.115	5.021	<.001	
Emotional stability	–0.182	–7.808	<.001	
Green zone				
Intercept	NA	3.630	<.001	0.137
Sex	0.153	2.885	.004	
Age	–0.174	–3.271	.001	
Need for cognitive closure	0.261	4.947	<.001	

Abbreviation: NA, not applicable.

^a^ Data are adjusted for sex and age.

## Discussion

This survey study explored the levels of knowledge, worries, risk perceptions, and preventive behaviors associated with the COVID-19 outbreak in Italy among respondents from areas with different degrees of exposure to the pandemic. Furthermore, we evaluated the associations of these concerns with the quality of life ratings to understand which individual characteristics were associated with these psychological and behavioral reactions.

We reached a relatively large sample in a short period of time, suggesting a desire from a part of the population to actively contribute to scientific research. Participants were well informed about the virus characteristics and most of the recommended behaviors, although they reported some confusion about the use of antibiotics and the possibility to contract SARS-CoV-2 by touching objects. We found that, after the first week since the beginning of the Italian outbreak, the level of worries was relatively low across all 3 zones, but the concern of contracting SARS-CoV-2 increased with the geographic proximity to the center of the outbreak, in line with previous findings.^19^ As expected, the same geographic pattern emerged in the adoption of precautionary measures. Interestingly, the reports of worries related to seeing other people scared by the possibility of contagion did not change across the zones. Similar to previous studies,^9,30^ we found that women reported higher levels of perceived risk and preventive behaviors. Furthermore, in our sample, young respondents were more sensitive to social appraisal and more inclined to adopt preventive behaviors than older respondents.

Concerning the associations of risk perception with quality of life, in our sample, physical and mental well-being were similar among the respondents from all 3 zones. Thus, both geographic proximity and risk perception about contracting COVID-19 did not seem to be significantly associated with the quality of life. However, we found that the perception of other people’s emotional worries, which included both beliefs about social restrictions and the fear of the illness itself, was associated with participants’ quality of life, confirming that the an individual’s appraisal of the way others evaluate a situation plays a crucial role for that individual facing many emotional events,^31^ regardless of the proximity to the contagion. This is true even where the geographic distance from the outbreak was not immediate. When in emergency situations, as was the case of the red zone, individual personality differences tend to be less informative of the worries and pandemic-related behaviors. However, the regression models were quite conservative, given the need to account for multiple comparisons, and the sample was unbalanced across areas. In fact, when examining the values of the correlations, some interesting associations were found in all 3 areas. We found different trends, varying by contagion zone, when considering the associations of the individual characteristics with worries and behaviors. On the one hand, in the red zone, worries about COVID-19 were associated with emotional stability, suggesting that people who were resilient (ie, had high emotional stability scores) experienced lower levels of concerns and fears than those who were more emotionally unstable. Moreover, in the red zone, openness was associated with a lower perceived susceptibility, and cognitive closure was associated with higher social appraisal. This suggests that people with a curious, flexible, and open attitude may be less influenced by other people’s behaviors. Low emotional stability was associated with risk perception in all 3 regions, which agrees with the fact that individuals who experience neurotic symptoms typically appraise stressors as more threatening and less controllable^32^ and display greater emotional reactivity in facing psychological stress^33^ than others. On the other hand, outside of the quarantine zone, the other relevant association was with the need for cognitive closure. Individuals scoring high on this trait tend to strive for closure by avoiding new information to more quickly reach closure and are more likely act to diminish uncertainty.^34^ Thus, results from this study confirm that a lower ability to tolerate ambiguity was associated with sensitivity to others’ emotional reactions and preventive behaviors to cope with the threat.^19^

According to our findings, seeing other people worried through contagion-related behaviors, especially when irrational (eg, depleting the grocery stores), was more associated with psychological well-being than were worries about the virus itself. According to the neurotic cascade hypothesis,^35^ the processes behind this emotional contagion may constitute a vicious cycle: individuals high in neuroticism are more likely to worry more about COVID-19–related information because they appraise this event as more harmful or threatening, and they also react with more stress responses. This construct supports the importance of implementing communication strategies that do not promote fear, anxiety, and insecurity, which would spread over social media and negatively impact the quality of life of individuals from all regions. Moreover, it is possible that those with a high propensity toward anxiety and insecurity could be more easily influenced by social communications.

These these findings suggest that helping people from the general population to become more tolerant of uncertainty and more adept at regulating their emotions may aid in preventing the development of virus-related worries. To achieve this goal, a good opportunity is represented by mindfulness, defined as nonjudgmental awareness of the present moment, with an open and flexible mindset, which has been found to be associated with a number of positive outcomes in behavioral regulation and mental health.^36^ Mindfulness-based interventions can be provided with online and mobile protocols,^37^ compatible with the need for social distance. Furthermore, to successfully help people cope with worries deriving from ambiguity and uncertainty in similar emergency situations, health and political authorities should make efforts to simplify communications, give clear rules, and discourage irrational behaviors.

### Limitations

This study has some limitations. The main limitations deal with our sample’s composition: in general, participants had a higher level of education than the overall Italian populace, which prevents a generalization to the whole population. Furthermore, the subsamples from the 3 zones had different sizes, and the red zone was a comparatively smaller sample than the other 2 zones. Another study limitation was the lack of representativeness of the sample owing to the snowball sampling and lack of response rate.

## Conclusions

The findings of this survey study suggest that during the first week after the beginning of the COVID-19 outbreak in Italy, people were well informed and ready to conform to the suggested behaviors. This is particularly true for those in the most exposed areas. However, people from our sample did not report high levels of feeling susceptible to COVID-19. Similarly, it appears that, in the first phase of the outbreak, quality of life was not significantly impaired. These results reflect only the first week since the beginning of the epidemic, as long-term quarantines are associated with worse mental health.^5^

We found different associations among the zones: specifically, in the quarantine area, where clear and restricted rules were implemented, individual characteristics played less of a role in people’s worries than they did in other areas. For the rest of the country, which was not quarantined, it seems that low emotional stability and mental rigidity as the need for a stable and predictable context were associated with more severe concerns, in particular for a socially mediated risk perception.

To our knowledge, this is the first study that explores the psychological features of the COVID-19 pandemic in a Western country. Our data were collected within the first week since the detection of the first Italian cases of COVID-19 and after the beginning of the countermeasures set by health authorities. As individual behavior is crucial to control the spread of this pandemic,^38^ understanding the psychological reactions in the first phases could inform intervention policies and the communication strategies by the authorities. This is particularly relevant in situations, such as the current COVID-19 pandemic, where no drugs or vaccines are yet available and the main way to contain the spread of the virus is to support changes in individuals’ behaviors and their compliance with health prescriptions.

## Notes

Supplement. **eTable 1.** Frequency Distributions of the Items Measuring Worries About COVID-19 **eTable 2.** Descriptive Statistics and Analysis of Variance of Each Measure for Sex and Age **eFigure.** Sample Distribution Across Zones
