# Supplementary material for: Knowledge, Concerns, and Behaviors of Individuals During the First Week of the Coronavirus Disease 2019 Pandemic in Italy
Source: JAMA Netw Open. 2020 Jul 24;3(7):e2015821. doi: 10.1001/jamanetworkopen.2020.15821 (PMC7382000; doi:10.1001/jamanetworkopen.2020.15821)
Supplement: Supplement. — eTable 1. Frequency Distributions of the Items Measuring Worries About COVID-19 eTable 2. Descriptive Statistics and Analysis of Variance of Each Measure for Sex and Age eFigure. Sample Distribution Across Zones [file jamanetwopen-3-e2015821-s001.pdf]

Supplementary Online Content

Pagnini F, Bonanomi A, Tagliabue S, et al. Knowledge, concerns, and behaviors of individuals during the first week of the coronavirus disease 2019 pandemic in Italy. *JAMA Netw Open*. 2020;3(7):e2015821. doi:10.1001/jamanetworkopen.2020.15821

eTable 1. Frequency Distributions of the Items Measuring Worries About COVID-19
eTable 2. Descriptive Statistics and Analysis of Variance of Each Measure for Sex and Age
eFigure. Sample Distribution Across Zones

This supplementary material has been provided by the authors to give readers additional information about their work.

© 2020 Pagnini F et al. *JAMA Network Open.*

eTable 1. Frequency Distributions of the Items Measuring Worries About COVID-19

Items measuring worries about COVID-19	1	2	3	4	5	6	7	N
1) How worried are you about the Coronavirus-related situation? (from 1, not worried at all, to 7, extremely worried)	23.32%	17.17%	20.11%	23.11%	12.90%	2.61%	0.78%	2830
2) What do you think are the odds of catching the Coronavirus? (from 1, highly unlikely, to 7, highly likely)	3.15%	14.46%	22.41%	28.31%	20.33%	8.27%	3.08%	2829
3) As for his social behavior. in the next few days... (from 1, I will try not to meet anyone. to 7, I will not limit contacts with other people)	2.01%	5.73%	13.08%	23.44%	20.43%	18.70%	16.61%	2829
4) In the next few days, will you physically distance yourself from the people you will meet? (from 1, absolutely yes, to 7, absolutely not)	2.09%	5.24%	11.72%	18.05%	21.84%	20.78%	20.28%	2825
5) Does seeing people around you worried about the virus increase your concern? (from 1, absolutely yes, to 7, absolutely not)	4.71%	7.50%	15.50%	15.04%	16.42%	17.45%	23.36%	2825
6) Does hearing about the virus in a worrisome way increase your perception of risk? (from 1, absolutely yes, to 7, absolutely not)	8.28%	11.68%	19.33%	11.79%	14.19%	15.36%	19.36%	2825
7) Does meeting people who experience a physical discomfort such as cough. sneazing. or similar. make you feel in danger? (from 1, absolutely yes, to 7, absolutely not)	4.67%	8.85%	18.65%	19.26%	18.69%	17.06%	12.81%	2825
8) Does seeing "panic scenes" in people around you (e.g.. spasmodic search for disinfectants, depleting supermarkets. etc.) increase your perception of risk? (from 1, absolutely yes, to 7, absolutely not)	4.53%	6.83%	14.86%	12.27%	15.99%	19.03%	26.49%	2827

© 2020 Pagnini F et al. *JAMA Network Open.*

eTable 2. Descriptive Statistics and Analysis of Variance of Each Measure for Sex and Age

Variables	min	max	N	gender		*p*	*eta*	age			*p*	*Effect size*
				M	F			18-24	25-40	>40		
Physical health	13	69	2619	54.54	54.11	0.10	0.032	54.87	54.53	52.22	<0.001	0.184
Mental health	10	70	2619	49.18	47.46	<0.001	0.069	46.71	47.37	51.14	<0.001	0.168
Optimism	1	5	2447	2.64	2.75	0.003	0.060	2.85	2.75	2.42	<0.001	0.209
Need for cognitive closure	1	6	2437	3.71	3.86	<0.001	0.077	3.90	3.78	3.70	<0.001	0.102
Internal locus of control	1	6	2411	3.89	3.69	<0.001	0.093	3.74	3.74	3.71	0.79	0.014
Agreeableness	1	5	2428	3.22	3.18	0.32	0.020	3.09	3.18	3.41	<0.001	0.150
Conscientiousness	1	5	2428	3.60	3.72	0.002	0.062	3.56	3.72	3.99	<0.001	0.209
Emotional Stability	1	5	2428	3.24	2.88	<0.001	0.149	2.83	2.98	3.26	<0.001	0.165
Extroversion	1	5	2428	3.16	3.25	0.03	0.042	3.21	3.28	3.20	0.25	0.034
Openness	1	5	2428	3.70	3.63	0.13	0.031	3.61	3.72	3.65	0.05	0.050
epidemic-related worries	1	7	2830	2.57	3.08	<0.001	0.146	2.95	2.97	2.99	0.80	0.012
perceived susceptibility	1	7	2829	3.60	3.93	<0.001	0.100	3.95	3.96	3.49	<0.001	0.140
preventive behaviors	1	7	2829	2.99	3.18	0.003	0.057	3.06	3.22	3.22	0.006	0.060
social appraisal	1	7	2828	2.95	3.52	<0.001	0.160	3.60	3.32	2.97	<0.001	0.164

eFigure. Sample Distribution Across Zones

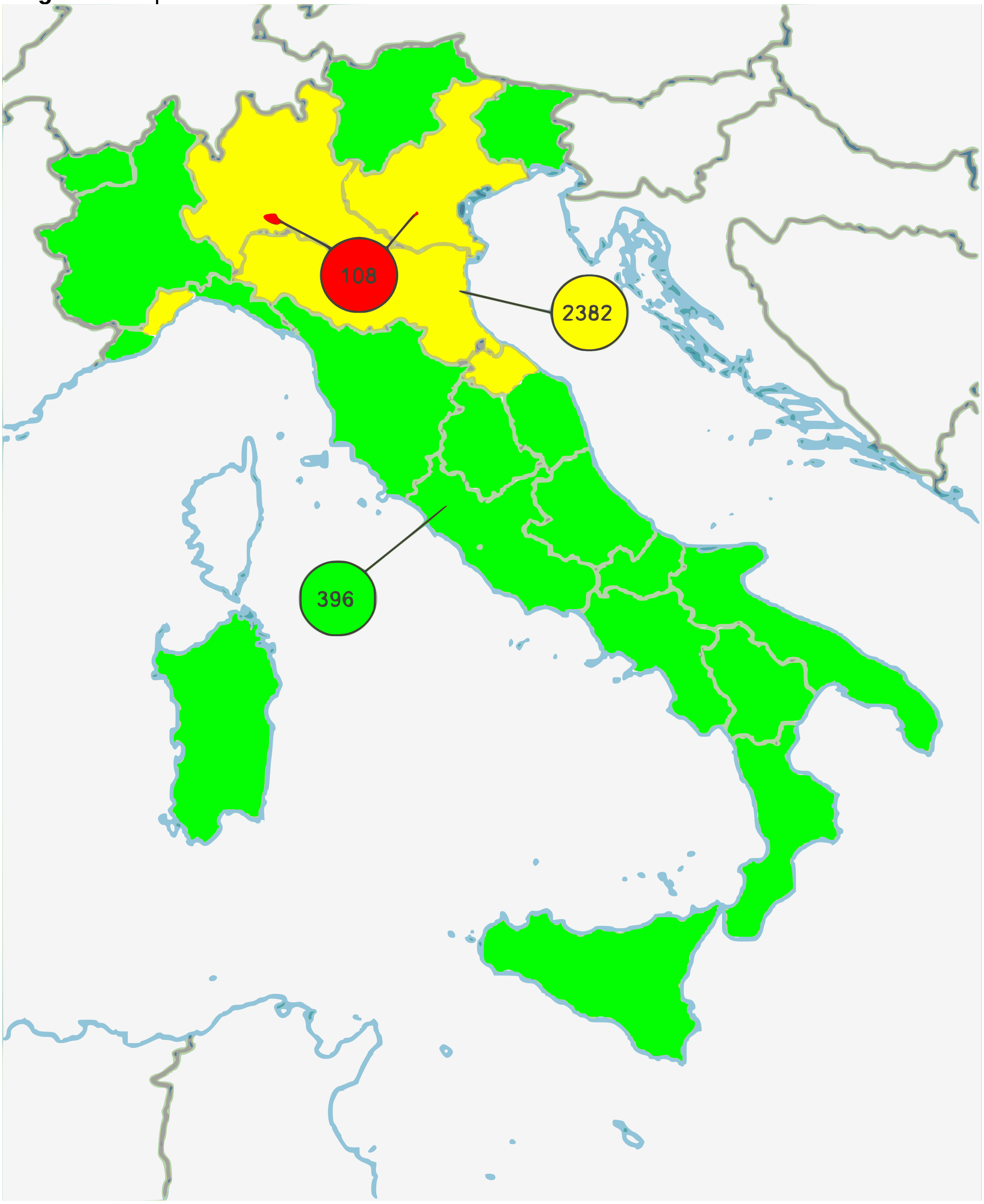

© 2020 Pagnini F et al. *JAMA Network Open.*
